# Digital Phenotyping Self-Monitoring Behaviors for Individuals With Type 2 Diabetes Mellitus: Observational Study Using Latent Class Growth Analysis

**DOI:** 10.2196/17730

**Published:** 2020-06-11

**Authors:** Qing Yang, Daniel Hatch, Matthew J Crowley, Allison A Lewinski, Jacqueline Vaughn, Dori Steinberg, Allison Vorderstrasse, Meilin Jiang, Ryan J Shaw

**Affiliations:** 1 School of Nursing Duke University Durham, NC United States; 2 Center of Innovation to Accelerate Discovery and Practice Transformation Durham Veterans Affairs Medical Center Duke University Durham, NC United States; 3 Division of Endocrinology, Diabetes and Metabolism School of Medicine Duke University Durham, NC United States; 4 College of Nursing New York University New York, NY United States; 5 Department of Biostatistics University of Florida Gainesville, FL United States; 6 Center for Applied Genomics and Precision Medicine School of Medicine Duke University Durham, NC United States

**Keywords:** digital phenotype, latent class growth analysis, type 2 diabetes, self-management, self-monitoring, Mobile Health

## Abstract

**Background:**

Sustained self-monitoring and self-management behaviors are crucial to maintain optimal health for individuals with type 2 diabetes mellitus (T2DM). As smartphones and mobile health (mHealth) devices become widely available, self-monitoring using mHealth devices is an appealing strategy in support of successful self-management of T2DM. However, research indicates that engagement with mHealth devices decreases over time. Thus, it is important to understand engagement trajectories to provide varying levels of support that can improve self-monitoring and self-management behaviors.

**Objective:**

The aims of this study were to develop (1) digital phenotypes of the self-monitoring behaviors of patients with T2DM based on their engagement trajectory of using multiple mHealth devices, and (2) assess the association of individual digital phenotypes of self-monitoring behaviors with baseline demographic and clinical characteristics.

**Methods:**

This longitudinal observational feasibility study included 60 participants with T2DM who were instructed to monitor their weight, blood glucose, and physical activity using a wireless weight scale, phone-tethered glucometer, and accelerometer, respectively, over 6 months. We used latent class growth analysis (LCGA) with multitrajectory modeling to associate the digital phenotypes of participants’ self-monitoring behaviors based on their engagement trajectories with multiple mHealth devices. Associations between individual characteristics and digital phenotypes on participants’ self-monitoring behavior were assessed by analysis of variance or the Chi square test.

**Results:**

The engagement with accelerometers to monitor daily physical activities was consistently high for all participants over time. Three distinct digital phenotypes were identified based on participants’ engagement with the wireless weight scale and glucometer: (1) low and waning engagement group (24/60, 40%), (2) medium engagement group (20/60, 33%), and (3) consistently high engagement group (16/60, 27%). Participants that were younger, female, nonwhite, had a low income, and with a higher baseline hemoglobin A_1c_ level were more likely to be in the low and waning engagement group.

**Conclusions:**

We demonstrated how to digitally phenotype individuals’ self-monitoring behavior based on their engagement trajectory with multiple mHealth devices. Distinct self-monitoring behavior groups were identified. Individual demographic and clinical characteristics were associated with different self-monitoring behavior groups. Future research should identify methods to provide tailored support for people with T2DM to help them better monitor and manage their condition.

**International Registered Report Identifier (IRRID):**

RR2-10.2196/13517

## Introduction

Sustained self-management with consistent self-monitoring is essential for individuals with type 2 diabetes mellitus (T2DM) to help them maintain optimal health [1]. Mobile health (mHealth) devices (eg, apps, Fitbit, Apple Watch, wireless scale, glucometer) are widely available and can help support engagement in T2DM self-management behaviors [2]. Using mHealth devices to monitor weight, blood glucose levels, activity levels, and dietary behaviors has proven to be feasible and effective in adults with T2DM [3-5]. Despite these benefits of mHealth tools, research indicates that engagement with mHealth tools decreases over time, and these trends also vary according to individual characteristics [6-10]. Determining these patterns of engagement with mHealth tools over time and how individual characteristics are associated with various patterns may provide crucial understanding on the use of mHealth tools to support T2DM self-monitoring and self-management.

Digital phenotyping, the concept of using data from mHealth devices to augment assessment of human illness, is rapidly emerging [11]. To date, digital phenotyping has been successfully used to track behavior and symptom data and to refine diagnosis and risk prediction for psychiatric disorders [12], dementia [13], and asthma [14]. Digital phenotyping has also been used to facilitate chronic disease management, such as using wearable accelerometers to track functional outcomes in patients with neurological disorders and to facilitate rehabilitation programs [15]. Although the actual readings or values from mHealth devices provide vital information for disease diagnosis, prognosis, and management, the engagement trajectories with multiple mHealth devices over time also provide crucial information about self-monitoring behaviors for patients with chronic diseases. Individuals who engage with mHealth devices more frequently indicate better self-monitoring behavior.

Latent class growth analysis (LCGA) is a type of growth mixture model that can determine individual phenotypes by identifying subgroups who follow similar trajectories over time on one or more outcomes. LCGA has been used extensively in the social sciences [16]. In medical and nursing research, LCGA has been used to quantify patient risk profiles based on physiological measures [17] and symptom research [18]. The method was extended by Jones and Nagin [19] to identify distinct subgroups based on trajectories across multiple outcomes.

In this study, we sought to demonstrate how to digitally phenotype the self-monitoring behaviors of individuals with T2DM based on their engagement trajectories with multiple mHealth devices using LCGA with multitrajectory modeling. Further, we explored if the participants’ digital phenotypes on self-monitoring behaviors varied according to their baseline demographic and clinical characteristics.

## Methods

### Design and Sample

This was a longitudinal observational feasibility study using multiple mHealth devices for patients with T2DM. The complete details of the study protocol were previously reported [5]. Sixty individuals with T2DM were recruited from a single primary care clinic and were followed for 6 months. As described in Table 1, participants were asked to perform self-monitoring using three measures on three mobile devices provided by the study over 6 months. This included weight (pounds) measured by a cellular-enabled scale provided by BodyTrace (Palo Alto, CA, USA), blood glucose (mg/dL) measured through a phone-tethered glucometer provided by iHealth (Mountain View, CA, USA), and physical activity measured in daily steps by a wrist-worn accelerometer and associated fitness app provided by Fitbit (San Francisco, CA, USA). Participants reported demographic information at baseline in Research Electronic Data Capture, a secure, web-based software platform designed to support data capture for research studies [20,21]. Daily monitoring on weight and physical activity were required by the study protocol, whereas blood glucose monitoring was prescribed by the primary care physician, which was performed at least once a week. Participants’ hemoglobin A_1c_ (HbA_1c_) values were extracted from their electronic health record from the closest date to baseline and 6 months postbaseline. Duke University’s Institutional Review Board approved all study activities (IRB No. Pro00071569).

**Table 1:** Mobile health devices used and time points for data collection.

Variable	Instrument	Time points
Weight (pounds)	Cellular-enabled Scale (BodyTrace)	Daily
Blood glucose (mg/dL)	Food and Drug Association-approved wireless glucometer (iHealth)	As prescribed by the primary care physician, at least once a week
Physical activity (number of steps)	Triaxial accelerometer and associated fitness app (Fitbit)	Daily
Hemoglobin A_1c_ (mmol/mol)	Electronic health record laboratory results	Baseline and 6 months postbaseline

### Measures

Self-monitoring behaviors were captured by engagement with different mHealth devices on tracking weight, blood glucose, and physical activity. We operationalized engagement with the wireless weight scale and accelerometer as the percentage of days that the participants used the devices during 13 biweekly periods over 6 months. Since some participants may measure blood glucose multiple times per day, we operationalized engagement with the glucometer as the percentage of days that participants measured blood glucose at least once a day

Covariates included age (years), gender, race (nonwhite, white), income (1=<US $10,000, 2=US $10,000-19,999, 3=US $20,000-29,999, 4=US $30,000-39,999, 5=US $40,000-49,999, 6=US $50,000-59,999, 7=US $60,000-79,999, 8≥US $80,000), insulin dependence (currently taking any insulin medication), and baseline HbA_1c_ values.

### Data Analysis

Descriptive statistics were used to summarize demographic and other participant characteristics at baseline, including age, gender, race, income, insulin dependence, and HbA_1c_ level. Empirical summary plots of biweekly engagement rates over 6 months were created for each device to illustrate the trajectories of self-monitoring behaviors for weight, blood glucose, and physical activity.

We conducted LCGA using SAS Proc Traj [19,22] to identify latent groups of trajectories in biweekly engagement over the 6-month observation period. We first modeled the trajectories of biweekly engagement of each device separately to determine the number of latent classes that offered the best fit for each device. Because engagement rate is a continuous variable with an approximately normal distribution, we used the censored normal distribution (cnorm). Based on the empirical summary plots, we tested both linear and quadratic trend models and chose the number of latent groups based on different number of groups using both the Akaike information criterion (AIC) and Bayesian information criterion (BIC) values in addition to clinical judgement of the study team. After modeling the trajectories for each device, we modeled the trajectories of adherence to the devices simultaneously using a multitrajectory model.

To examine the relationships between participant characteristics, clinical variables, and latent trajectory group membership, we conducted analysis of variance (ANOVA) for age, income level, and baseline HbA_1c_ value, and Chi square tests for race, gender, and insulin dependence. Finally, ANOVA was conducted to assess if a latent trajectory group identified in the LCGA multitrajectory model was associated with 6-month HbA_1c_ values and changes in HbA_1c_ from baseline to 6 months. All data analyses were conducted with SAS 9.4 (Cary, NC, USA).

## Results

### Sample

Demographic characteristics are presented in Table 2. The majority of the participants were women and nonwhite. The median income was US $40,000-49,999, and approximately half of the participants were currently using insulin medication. More detailed information on the sample and recruitment was reported previously [10].

**Table 2:** Demographic characteristics (N=60).

Characteristic		Value
Age, mean (SD)		55.1 (11.7)
Gender: Female, n (%)		43 (72)
Race: Black/Non-White, n (%)		39 (65)
**Income level (USD), n (%)**		
	<$10,000	4 (7)
	$10,000- 19,999	3 (5)
	$20,000- 29,999	8 (14)
	$30,000- 39,999	5 (9)
	$40,000- 49,999	11 (20)
	$50,000- 59,999	6 (11)
	$60,000- 79,999	5 (9)
	≥$80,000	14 (25)
**Insulin dependent, n (%)**		
	Yes	29 (48)
	No	31 (52)
Hemoglobin A_1c_ at baseline, mean (SD)		8.1 (1.8)

### Engagement Trajectories and Self-Monitoring Behavior Phenotypes

**Figure 1:**
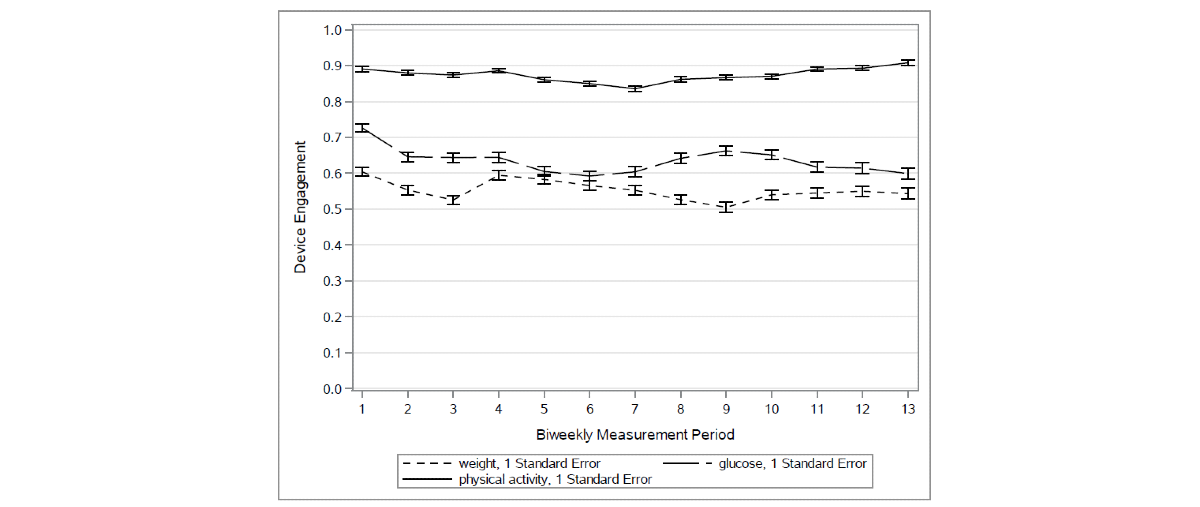
Empirical plots (mean, SEM) for biweekly engagement trajectories for each mobile health device over all 6 months.

Empirical summary plots of engagement with different mHealth devices are presented in Figure 1 to show overall engagement trends. The average engagements with the wireless scale and glucometer for all participants were moderate (52%-72%) and showed a slightly decreasing trend for the first 2 weeks. In contrast, engagements with the accelerometers remained high over time at around 90% and with very minimal variability across all participants.

**Figure 2:**
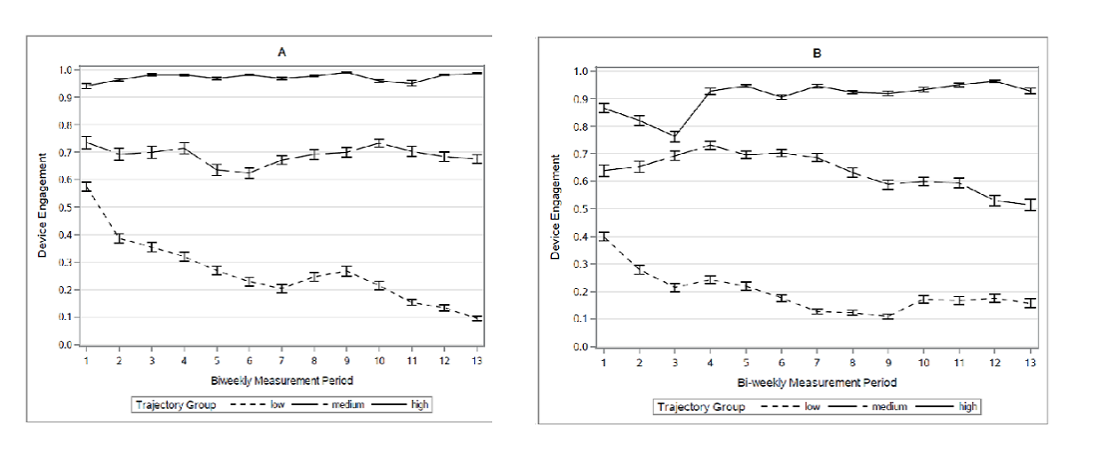
Empirical summary plot for biweekly engagement trajectories with the (A) glucometer and (B) wireless weight scale by different engagement groups.

Three latent classes of engagement trajectories were identified (Table 3, Figure 2): low and waning engagement group, medium engagement group, and consistently high engagement group. The AIC and BIC values of this model were –825.1 and –846.0, respectively. In the low engagement group, individuals had relatively lower engagement with daily weight and glucose monitoring at baseline (40% and 56%, respectively) and showed a statistically significant decrease in daily weight and glucose monitoring over time. The drop in engagement with daily weight monitoring was faster in the first 2 weeks and was captured by a significant quadratic term. The moderate engagement group showed moderate engagement with daily weight and glucose monitoring at baseline (65% and 72%, respectively) and no statistically significant change over time. In the high engagement group, high engagement with daily weight and glucose monitoring at baseline was observed (82% and 94%, respectively). In this group, a slight but statistically significant increase in weight monitoring was observed over the 6 months, whereas glucose monitoring did not change. The final three-class model was chosen based on a model fit procedure according to AIC and BIC values (Table 4). For all devices, model fit was improved in the three-class model compared to the two-class model. However, for weight and glucose, fit improved only marginally in the four-class model relative to the three-class model. For physical activity, the four-class model could not be produced. The final three-class models were based on weight and glucose device engagement trajectories because the engagement rate for the physical activity device was consistently high over time for all participants with very small variabilities.

**Table 3:** Latent class growth analysis multitrajectory model results for engagement with a wireless weight scale and glucometer (N=60).

Variable			B	SE	*t*_1_	*P* value
**Group membership**						
	Low Engagement (n=24)		40%	6.40	6.21	<.001
	Medium Engagement (n=20)		33%	6.30	5.27	<.001
	High Engagement (n=16)		27%	5.91	4.57	<.001
**Weight**						
	**Low Engagement**					
		linear	–0.09	0.02	–3.67	<.001
		quadratic	0.005	0.002	2.82	.005
	**Medium Engagement**					
		linear	0.02	0.03	0.71	.48
		quadratic	–0.003	0.002	–1.41	.16
	**High Engagement**					
		linear	0.02	0.008	2.34	.02
**Glucose**						
	**Low Engagement**					
		linear	–0.07	0.03	–2.27	.02
		quadratic	0.002	0.002	0.90	.37
	**Medium Engagement**					
		linear	–0.02	0.03	-0.62	.54
		quadratic	0.001	0.002	0.60	.55
	**High Engagement**					
		linear	–0.003	0.01	-0.27	.79

**Table 4:** Model fit by number of latent classes modeleda.

Number of classes	Weight			Glucose			
	AIC^b^	BIC^c^	Percent per class		AIC	BIC	Percent per class
2	–420.1	–427.4	48.7/51.3		–457.4	–464.7	56.3/43.7
3	–368.4	–379.9	39.3/20.8/39.9		–408.3	–419.8	45.1/25.0/29.8
4	–352.2	–368.0	8.7/32.4/19.1/39.7		–393.3	–409.0	29.0/16.7/24.4/29.9

^a^Sample size per class is based on most likely class membership.

^b^AIC: Akaike information criterion

^c^BIC: Bayesian information criterion.

### Associations of Self-Monitoring Behavior Phenotypes With Participant and Clinical Characteristics

The results of bivariate analyses examining how self-monitoring phenotypes are related to participant characteristic are presented in Table 5. Self-monitoring phenotype was significantly associated with age, in that those in the low and waning engagement group were younger compared to those in the medium and consistently high engagement groups. Self-monitoring phenotype was also significantly related to gender as there were less women in the high and medium engagement groups compared to the low engagement group. Race was also significantly associated with group membership, in that those in the low engagement group were more likely to be nonwhite than those in the high engagement group. Insulin dependence was not significantly associated with engagement group.

Table 6 summarizes the association between the self-monitoring behavior phenotypes and HbA_1c_ at baseline, 6 months, and the change between baseline and 6 months. Although the change in HbA_1c_ from baseline to 6 months did not differ according to engagement group, the low engagement group had higher baseline HbA_1c_ values compared to those of the medium and high engagement groups. This trend continued at the 6-month follow up, with higher HbA_1c_ values in the low engagement group compared to the medium and high engagement groups.

**Table 5:** Bivariate relationships between baseline demographic and clinical characteristics and engagement group membership.

Variable			Low Engagement (24/60, 40%)	Medium Engagement (20/60, 33%)	High Engagement (16/60, 27%)	Test statistic	*P* value
Age, mean (SD)			49.1 (13.0)	57.9 (10.5)	60.6 (6.3)	*F*_2, 57_= 6.48	.003
Gender: Female, n (%)			20 (47)	10 (23)	13 (30)	χ^2^_2_=6.96	.03
Income^a^, mean (SD)			4.3 (2.2)	5.7 (2.0)	5.9 (2.3)	*F*_2, 53_=3.13	.05
Race: Black/Non-White, n (%)			20 (51)	11 (28)	8 (21)	χ^2^_2_=6.01	.05
**Insulin dependent, n (%)**						χ^2^_2_=1.80	.41
	Yes		14 (48)	9 (31)	6 (21)		
	No		10 (33)	11 (36)	10 (32)		
Hemoglobin A_1c_ at baseline, mean (SD)			9.01 (2.13)	7.61 (1.17)	7.34 (1.16)	*F*_2,56_=6.30	.003

^a^Income categories: 1=< $10,000, 2=$10,000-19,999, 3=$20,000-29,999, 4=$30,000-39,999, 5=$40,000-49,999', 6=$50,000-59,999, 7=$60,000-79,999, 8≥'$80,000.

**Table 6:** Hemoglobin A_1c_ levels (mean, SD) at baseline, 6 months, and change according to multitrajectory engagement group (N=60).

Time point	Low Engagement (n=24)	Medium Engagement (n=20)	High Engagement (n=16)	*F*	df	*P* value
Baseline	9.01 (2.13)	7.61 (1.17)	7.34 (1.16)	6.30	2,56	.003
Six months	8.64 (2.54)	7.09 (1.45)	7.16 (1.23)	3.96	2,49	.03
Change from baseline to 6 months	0.0 (2.23)	–0.44 (1.07)	–0.19 (0.64)	0.38	2,48	.68

## Discussion

Consistent self-monitoring and self-management of T2DM improves health outcomes [1,23,24]. Given the growing popularity of using mHealth devices to facilitate self-monitoring, engagement with mHealth devices has become an important tool to develop digital phenotypes based on individuals’ self-monitoring behaviors. This study is among the first to operationalize digital phenotyping of self-monitoring behaviors by applying LCGA modeling on engagement trajectory data from multiple mHealth devices.

Overall, individuals’ engagement with an accelerometer to monitor daily physical activities was consistently high (>82%) for the participants over time. Similar patterns were observed in other mHealth studies [25,26]. This may be due to the passive nature of data collection and transmission of these devices. We were able to identify three distinct digital phenotypes of self-monitoring behaviors using engagement trajectories of wireless weight scales and glucometers. There was a low and waning engagement group (24/60, 40%), a medium engagement over time group (20/60, 33%), and a consistently high engagement group (16/60, 27%). Specifically, the low engagement group started with low engagement in using both the wireless scale (40%) and glucometer (58%), and then the level of engagement rapidly decreased in the first 2 weeks.

These results are similar with those of other studies focused on the use of mHealth technologies or devices for chronic disease management [7]. However, our study provides further evidence by identifying individuals with low engagement in the first couple weeks, demonstrating the need to allocate additional intervention or resources since it is likely that waning engagement will be observed over time. For people who are highly engaged initially, we could consider providing minimum support to save resources as they will be more likely to stay engaged over time.

Our findings also demonstrate how engagement with mHealth devices varies according to patient demographic and clinical characteristics. The individuals in the high and moderate engagement groups were older, included more men, had higher income levels, were more likely to be white, and had better HbA_1c_ values at baseline. By contrast, the low and waning engagement group members were younger, included more women, had lower incomes, were more likely to self-identify as black or nonwhite, and had poorer control of their T2DM. Participants who are insulin-dependent may be required to self-monitor their blood glucose daily or even multiple times a day based on instructions from their primary care physician. This will certainly increase the motivation to engage with the glucometer or even wireless scales for the study participants. However, we did not find any significant association between insulin dependence and engagement group. This implies that we may need to provide further support to this high-risk group. The baseline characteristics of our sample are similar to those of prior research in that lower income individuals, nonwhite individuals, and women face more challenges in controlling glycemia, experience more T2DM complications, and have higher mortality rates [6,27]. We hypothesize that the younger patients in our study may have had lower engagement due to competing demands on their time (eg, caring for family, work), more comorbidities, or having been diagnosed with diabetes at a younger age. Digital phenotypes of self-monitoring behaviors can identify patients who may need the most support in changing health care behaviors and can inform strategies to tailor the use of mHealth tools in the delivery of self-management interventions. This result also indicates that different retention approaches may be needed for certain populations to maintain engagement with mHealth tools in support of T2DM self-management.

As discussed above, individuals with well-controlled baseline HbA_1c_ were more likely to be in the consistently high engagement group. Not surprisingly, these individuals continued to have better controlled HbA_1c_ at the 6-month follow up. However, the change in HbA_1c_ value between baseline and the 6-month follow up did not differ according to different phenotypes of self-monitoring behaviors or engagement groups. This indicates that good self-monitoring behaviors through active engagement with mHealth devices is helpful in maintaining well-controlled HbA_1c_, but does not necessarily further reduce HbA_1c_.

Limitations to the study include that the sample was obtained from a single site in the southeastern United States, which may not be representative of all patients with T2DM. A larger-scale study that includes more patients from different regions would yield more generalizable findings to a broader population. Such a study would also help to identify more complex patterns in engagement trajectories and more specific strategies in delivering behavior change interventions. Self-monitoring also occurred for only 6 months, which did not allow for examination of long-term patterns in a complex chronic illness such as T2DM. There are several factors that may affect a patient’s motivation to engage with the device and self-monitoring that was not accounted for in our analysis. First, this was an observational study and the participants were provided with different mHealth devices, which they could keep if they completed the study. Although we did not have any specific requirement or incentive for participants to use the device during the follow-up time, the ability to keep the device may have some implications in retaining their participation in the study. Second, during the 6-month follow-up period of the study, we conducted 20 interviews with the participants to view their data and gain perspectives on using real-time data collections to support self-monitoring. This may have also potentially affected the motivation for participants to engage.

In conclusion, T2DM is a challenging disease that requires frequent self-monitoring and consistent self-management. Digital phenotyping on self-monitoring behaviors using LCGA can help to identify subgroups of individuals with distinct engagement trajectories. Future research should focus on methods to develop tailored mHealth interventions based on the influence of different phenotypes of individuals on their self-monitoring behaviors.

## Abbreviations

AIC: Akaike information criterion
ANOVA: analysis of variance
BIC: Bayesian information criterion
HbA_1c_: hemoglobin A_1c_
LCGA: latent class growth analysis
mHealth: mobile health
T2DM: type 2 diabetes mellitus
